# Validation of angiography-derived Murray law-based quantitative flow reserve (μQFR) against pressure-derived instantaneous wave-free ratio for assessing coronary lesions, a single-center study in Egypt

**DOI:** 10.1186/s43044-024-00541-y

**Published:** 2024-08-26

**Authors:** Amir Anwar Samaan, Amir Mostafa, Sherif Lotfy Wahba, Matteo Kerlos, Ahmed Adel Elamragy, Khaled Shelbaya, Yasmin Elsobky, Mohamed Hassan

**Affiliations:** 1 Cardiology Department, Cairo University, Giza, Egypt; 2 Cardiology Department, AlNas Hospital, Qalyubia, Egypt; 3 Research Department, AlNas Department, Giza, Egypt

**Keywords:** Murray, Quantitative flow reserve, Functional significance

## Abstract

**Background:**

Instantaneous wave-free ratio (iwFR) is a well-validated method for functional evaluation of intermediate coronary lesions. A recently developed Murray law-based QFR (µQFR) allows wire-free FFR estimation using a high-quality single angiographic projection. We aim to determine the diagnostic accuracy of µQFR as compared to wire-based iwFR for physiological assessment of coronary lesions in a sample of Egyptian patients.

**Results:**

Over a one-year period, patients who previously underwent iwFR assessment of an intermediate coronary stenosis (40–90%) were retrospectively included. μQFR analysis was then performed offline using a dedicated artificial intelligence (AI)-aided computation software. All the measurements were performed blinded to iwFR results, and the agreement between iwFR and μQFR values was tested. Forty-nine patients (mean age 57.9 ± 9 years, 72.9% males) were included. Mean value of iwFR and μQFR was 0.90 ± 0.075 and 0.79 ± 0.129, respectively. There was a significant moderate positive linear correlation between μQFR and iwFR (*r* = 0.47, *p* = 0.001; 95% CI 0.22–0.68) with moderate-to-substantial agreement between the two methods (Kappa 0.6). In assessing the diagnostic accuracy of μQFR, the receiver operating characteristic (ROC) curve yielded an area under the curve (AUC) of 0.84 (95% CI 0.717–0.962) for predicting functionally significant lesions defined as iwFR < 0.89. The sensitivity and specificity of μQFR < 0.8 for detecting physiological significance of coronary lesions were 89% and 74% with positive and negative predictive values of 70 and 91%, respectively.

**Conclusion:**

µQFR has good diagnostic accuracy for predicting functionally significant coronary lesions with moderate correlation and agreement with the gold standard iwFR. Angiography-derived µQFR could be a promising tool for improving the utilization of physiology-guided revascularization.

## Background

A large body of evidence supports the use of functional coronary assessment to guide coronary revascularization decisions [1, 2]. Currently, either fractional flow reserve (FFR) or instantaneous wave-free ratio (iwFR) is considered the invasive gold standard for physiological evaluation of intermediate coronary stenoses (40–90%) [3]. Unfortunately, physiology-guided revascularization is still underutilized in daily practice in spite of current strong recommendations [4–6]. The real-world rate of FFR/iwFR use increased slowly from 14.8 to 18.5% in patients with intermediate coronary lesions [7]; however, the rate is significantly lower (less than 6%) in developing countries [8].

The presumed additional time and cost, the need for additional wire manipulation, and the discomfort experienced by some patients on inducing coronary hyperemia are the main reported reasons for the under usage of invasive coronary functional assessment [9]. This emphasizes the need for more convenient angiography-based, and wire-free method to increase the implementation of coronary physiology guidance.

Over the past years, several angiography-based methods for FFR estimation have been developed to overcome some of the limitations of wire-based FFR/iwFR. Two-dimensional quantitative coronary angiography (2D-QCA) was the first angiography-based tool to be used, yet it showed only a modest correlation with FFR [10]. Three-dimensional QCA was then introduced and demonstrated a higher diagnostic accuracy and correlation with FFR compared with the former. In 3D-QCA, computational fluid dynamics are applied to estimate the pressure drop across a stenotic segment. The process has been markedly advanced over years and got simplified to the point of providing a highly accurate FFR estimate in a few seconds [11–13].

Out of all commercially available 3D-QCA technologies, quantitative flow ratio (QFR) is the most known and widespread. It has been validated and proven to have an excellent accuracy to estimate FFR [14, 15]. However, the analysis requires two orthogonal or separate angiographic views with good quality that may not be applicable in some cases. Thus, in retrospective studies, about 15% of cases were not feasible for QFR analysis [16, 17]. In order to overcome this limitation, a new computational method that allows FFR estimation using a single high-quality angiographic projection (Murray law-based QFR, µQFR) has recently been developed and proved to predict clinical outcome [18, 19]. The aim of our work was to determine the diagnostic accuracy of this angiography-based, and wire-free µQFR method, as compared to the gold standard method (iwFR) in evaluation of angiographically intermediate coronary stenoses.

## Methods

### Study design and patient population

This is a single-center, observational, retrospective study conducted in ALNAS hospital. The study protocol was approved by ALNAS hospital institutional review board. Patients who underwent functional assessment of at least one native angiographically intermediate coronary stenosis using invasive iwFR in the interval between January 2023 and January 2024 were included. Intermediate coronary stenosis was defined as angiographic diameter stenosis between 40 and 90% based on the definition used in prior FFR and QFR studies, and as recommended by ESC guidelines on myocardial revascularization [3, 20]. Patients with aorto-ostial lesions, previous coronary artery bypass grafts (CABG), coronary aneurysm of the assessed vessel, lack of angiographic projection which is satisfactory for μQFR analysis were excluded. All these lesions were re-assessed for functional significance using μQFR.

### Clinical data

Demographic data were collected, and the presence of risk factors for atherosclerosis such as diabetes mellitus (DM), hypertension, dyslipidemia, or smoking was assessed. The clinical presentation and echocardiographic data were also collected.

### Invasive iFR measurement

Coronary angiography was performed according to standard local practice. The diagnostic catheter was exchanged for a guiding catheter with no side holes. Zeroing of the aortic pressure was done, and then, the *Volcano Verrata Plus pressure guide* wire was connected to the console. After calibration, the wire was then advanced into the coronary artery until the pressure sensor is positioned precisely at the end of the guide catheter that was flushed with saline at this point (due to the viscosity of contrast agent), and if necessary, disengaged from the coronary artery ostium. Pressure equalization between the wire and aortic pressure was performed. The pressure sensor of the wire was positioned directly distal to the lesion that is to be investigated in the main vessel, and not in a side branch. In case of sequential stenoses within one artery, the pressure sensor was placed downstream of the most distal lesion. An iwFR value of ≤ 0.89 was taken to indicate physiologically significant coronary stenosis. If the iwFR value was between 0.87 and 0.93, FFR was also performed to confirm the results.

### μQFR analysis

For each vessel, all projections were examined to select the optimal projection for the μQFR evaluation that avoid foreshortening, and overlap from other vessels. Two experienced operators independently assessed all the angiography images and excluded vessels with severe overlapping, tortuosity, foreshortening, and poor vessel opacification. In the event of disagreement, a third opinion was sought for consensus.

**Fig. 1:**
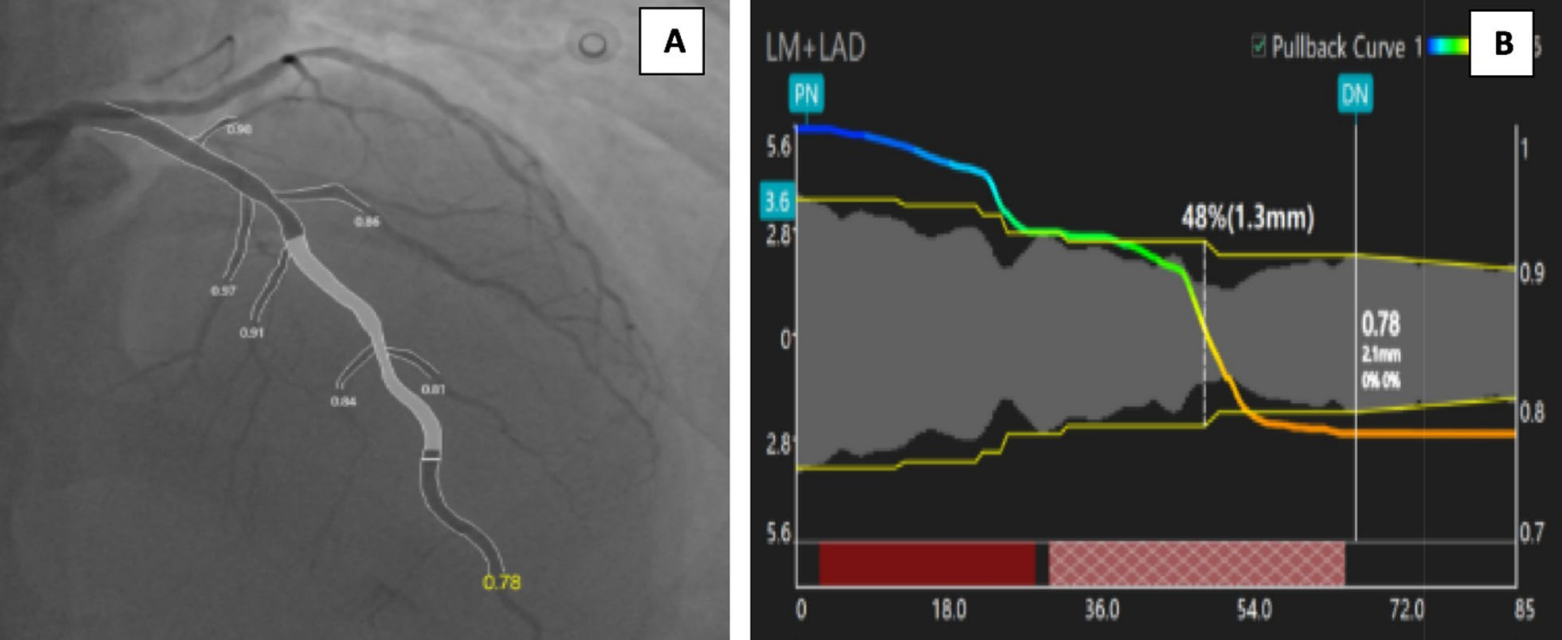
μQFR analysis of a lesion in the left anterior descending artery (LAD). **A** μQFR value of the assessed lesion, **B** μQFR values for MLD, MLD, maximal area stenosis, and length and diameter of proposed stent

μQFR analysis was performed offline using dedicated artificial intelligence (AI)-aided computation software (AngioPlus Core Pulse Medical Technology, Shanghai, China). This validated AI algorithm allows automatic delineation of lumen contours of major epicardial coronary arteries including side branches. Calculation of contrast flow velocity based on the length divided by the contrast dye-filing time was performed. A step-down reference diameter function was then reconstructed taking into account the flow going out through the side branches to calculate both the reference diameters and the pressure drop along the stenotic segment, according to Murray’s fractal law [21]. μQFR was calculated based on the selected angiographic projection, and blinded to iwFR results. Other parameters that were also obtained from the analysis included the vessel profile as minimal lumen diameter (MLD), reference diameter at MLD, and maximal area stenosis. In addition, data about PCI planning were obtained including the length and diameter of proposed stent in case of significant lesions **(**Fig. 1). A μQFR cutoff value of 0.8 was used to consider the lesion being physiologically significant [14, 18, 22].

### Statistical analysis

Baseline characteristics were summarized using numbers and percentages for categorical variables, mean and standard deviations for normally distributed continuous variables, and median and interquartile range for non-normally distributed continuous variables. A comparative analysis was conducted between the two modalities: iwFR, established as the standard of care, and μQFR, the test under investigation. Several statistical analyses were employed to assess the performance and concordance of these modalities.

Firstly, the reproducibility between iwFR and μQFR to determine the coronary lesion significance based on the commonly used cutoff value was determined through Cohen’s Kappa test. As a sensitivity analysis, Bland and Altman diagram was used to display the differences between the pairs of numerical readings.

Additionally, receiver operating characteristic (ROC) curve analysis was conducted to assess the discriminatory power of μQFR using iwFR value of ≤ 0.89 as the gold standard for diagnosing physiologically significant coronary stenosis. Furthermore, sensitivity and specificity analyses were performed to assess the diagnostic accuracy of μQFR by using each of the pre-specified μQFR cutoff value (0.8), and the best cutoff value resulted from the ROC curve.

Finally, Spearman correlation analysis was utilized to evaluate the strength and direction of the linear relationship between iwFR and μQFR measurements. Stata’s LOWESS (locally weighted scatterplot smoothing) function was used to assess the linearity between two variables visually.

Forty-three lesions are required to test the hypothesis of having an accuracy of at least 80% using a one-sample ROC analysis and a power of 80%, assuming that the prevalence of significant lesions will be 27%. After adding 15% for possible poor quality studies, 49 lesions are needed to be included. The sample size was calculated using Linden A. (2022). POWER ONEROC: Stata module to compute power and sample size for a one-sample ROC analysis.

## Results

A total of 49 patients were enrolled. The demographic and clinical characteristics of those patients are summarized in Table 1. The mean age was 59.7 years, with a standard deviation (SD) of 9 years. The majority of patients were males, accounting for 72.9% of the cohort. Hypertension was prevalent among 64.6% of study patients. Additionally, 56.3% of patients had DM, and 45.8% were smokers. Chronic kidney disease (CKD) was present in 20.8% of the cohort. The mean left ventricular ejection fraction (LVEF) was 53.96 ± 11.1%.

**Table 1:** Baseline characteristics of study patients

Variable	
Age, years	59.7 ± 9
Male gender	35 (72.9)
Smoking	22 (45.8)
Hypertension	31 (64.6)
DM	27 (56.3)
CKD	10 (20.8)
LVEF, %	54 ± 11.1 (45–60)
Clinical presentation	
ACS	6 (12)
CCS	38 (78)
ICM	5 (10)
Number of diseased vessels	
Single vessel	17 (33.3)
Multi-vessel	32 (66.7)
Culprit Vessel	
Distal LMT-LAD	1 (2)
LAD	32 (66)
LCX	8 (16)
RCA	8 (16)
Revascularization	
PCI	19 (39)
CABG	3 (6)
Deferred	27 (55)

Data are presented as no (%), mean ± SD

Indications for coronary angiography varied, with 12.5% (6 patients) presenting with acute coronary syndrome (ACS), 77.1% (38 patients) with chronic coronary syndrome (CCS), and 10.4% (5 patients) with ischemic cardiomyopathy (ICM). Among the study patients, 33.3% (16 patients) had single-vessel disease, while 66.7% (32 patients) had multi-vessel disease (Table 1).

Regarding the culprit vessel, the left anterior descending artery (LAD) was the most frequently involved, observed in 68% of cases, followed by the left circumflex artery (LCX) and right coronary artery (RCA), each accounting for 16% (8 patients). One case has distal LMT stenosis. In terms of revascularization procedures, 39% (19 patients) underwent percutaneous coronary intervention (PCI), and 6% (3 patients) underwent CABG, while revascularization was deferred in 27 (55%) of patients (Table 1).

**Fig. 2:**
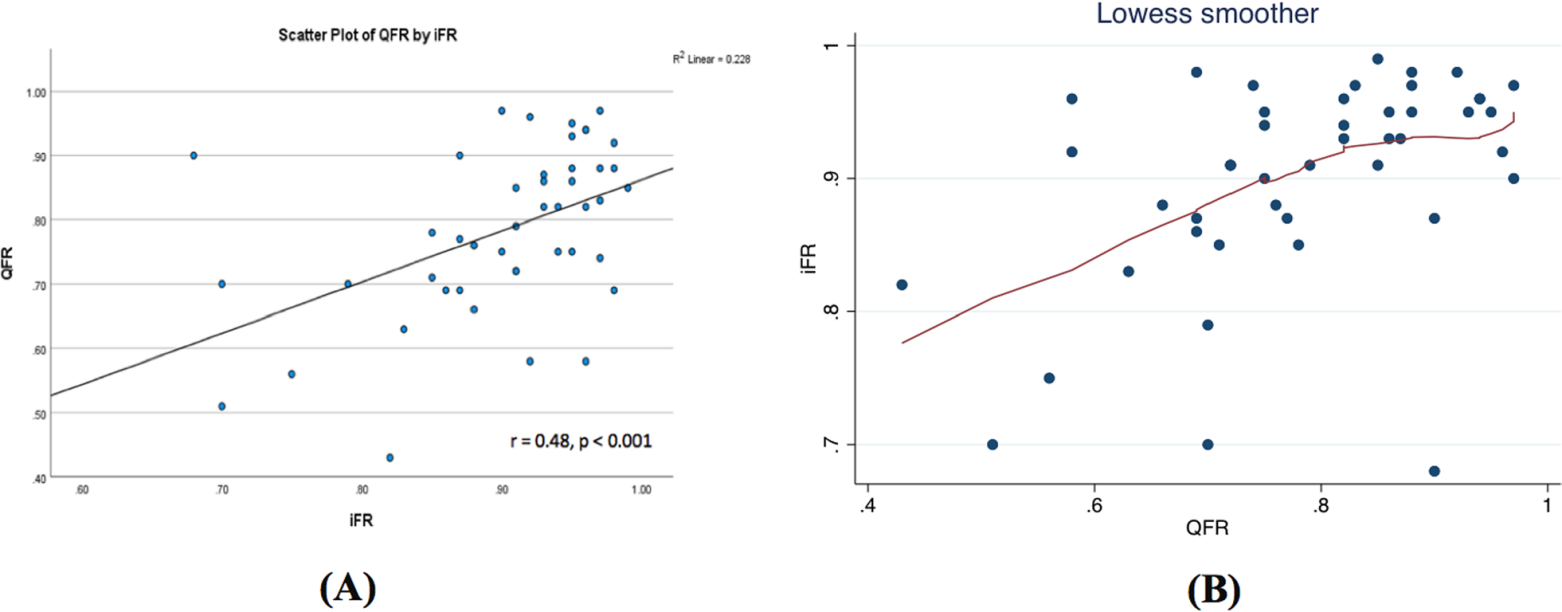
Correlation analysis (**A**) and linearity (**B**) between *μ*QFR and iwFR

**Fig. 3:**
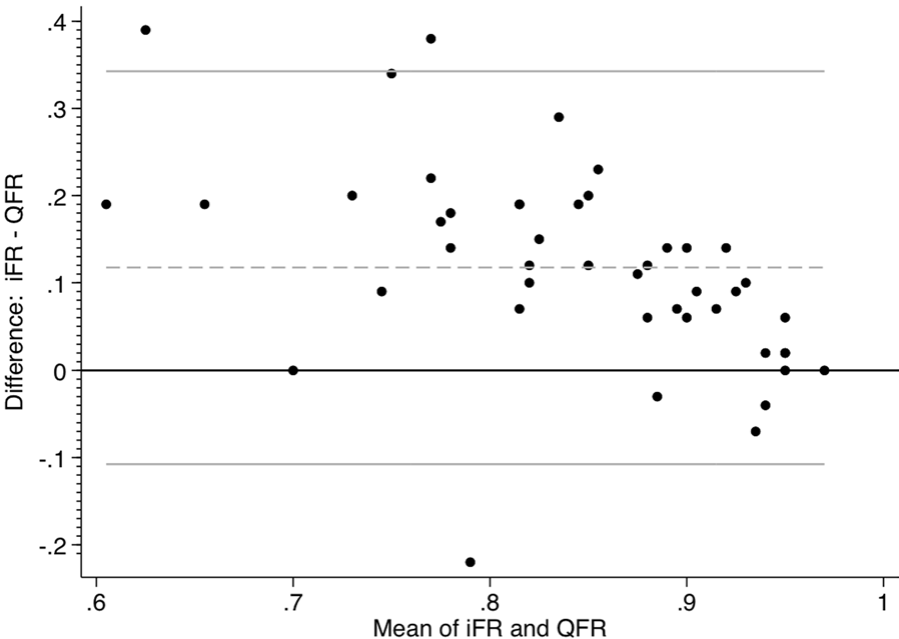
Bland and Altman Plot showing upper and lower limits of agreement between μQFR and iwFR

**Fig. 4:**
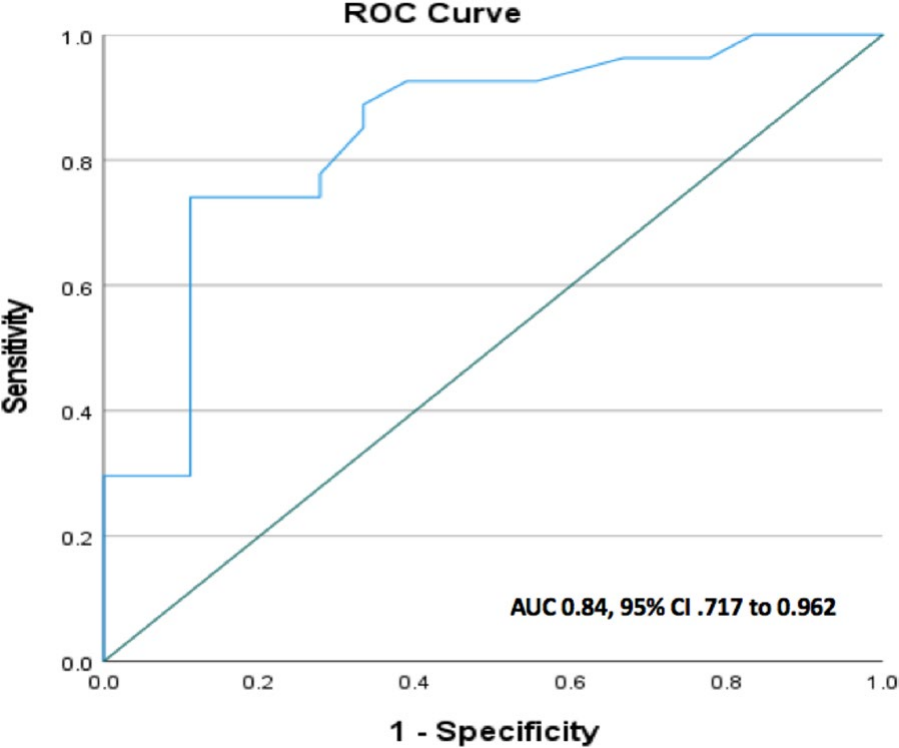
Receiver operating characteristic (ROC) curve demonstrating the diagnostic accuracy of μQFR based on iwFR significance (iwFR < 0.89)

iwFR was performed in all study patients (49 patients) with a mean value of 0.90 ± 0.075. FFR measurements were obtained in 9 patients who has iwFR value in the gray zone. μQFR was performed in 45 patients resulting in a mean value of 0.79 ± 0.129. μQFR measurement was not feasible in four patients due to vessel overlap and foreshortening. So, the diagnostic performance analyses were done on 45 coronary lesions only.

(A) Correlation and linearity of relationship between μQFR and iwFR
  There was significant moderate positive linear correlation between μQFR and iwFR (*r* = 0.47, *p* = 0.001; 95% CI 0.22–0.68). The relation between μQFR and iwFR slightly loses the linear relationship among the higher values (non-significant stenosis) (Fig. 2).
(B) The reproducibility between μQFR and iwFR
  There was a moderate-to-substantial agreement between μQFR and iwFR (Kappa 0.6) (Table 2), which was also displayed in the Bland and Altman diagram. The limits of agreement (reference range for difference) were − 0.112 to 0.347, and mean difference was 0.118 (CI 0.003 to 0.152) (Fig. 3).
(C) μQFR Performance: In assessing the diagnostic accuracy of μQFR, a receiver operating characteristic (ROC) curve was constructed, yielding an area under the curve (AUC) value of 0.84, with a 95% CI ranging from 0.717 to 0.962 (Fig. 4).

**Table 2:** Agreement between μQFR and iFR

		μQFR	
		Significant (*n* = 22)	Not significant (*n* = 23)
iwFR	Significant (*n* = 27)	20 (74.1%)	7 (25.9%)
	Not significant (*n* = 18)	2 (11.1%)	16 (88.9%)

**Table:** 

Agreement	Expected agreement	Kappa	Std. Err	*P* value
80%	50%	0.60	0.1454	< 0.001

In the evaluation of μQFR performance, the sensitivity, representing the true positive rate, was found to be 89%. Conversely, the specificity, or true negative rate, stood at 74% when we used either the best-cut value derived from the ROC curve, which is 0.82, or the pre-specified cutoff 0.80. Meanwhile, the positive predictive value (PPV) and negative predictive value (NPV) were 70% and 91%, respectively.

## Discussion

Physiological assessment of coronary stenosis, when added to angiographic appraisal, has been demonstrated to enhance the accuracy of diagnosis, optimize the selection of best management strategy, and improve clinical outcome. Unfortunately, and despite the current guidelines’ recommendations [3], the application of invasive iwFR and FFR is still limited in daily clinical practice especially in developing countries. The low adoption rates of physiology-guided revascularization strategies might be related to the cost of devices and drugs, prolonged procedure time, and additional coronary instrumentation, patient discomfort with administration of adenosine administration during FFR measurement, and limited confidence with the results, and experience. To the best of our knowledge, this is the first study in Egypt to validate angiography-derived μQFR against the gold standard iwFR in patients with intermediate coronary stenosis.

The current study demonstrated good diagnostic accuracy for μQFR in predicting hemodynamically significant coronary lesions (defined as iwFR < 0.89, and FFR < 0.8) with a moderate-to-substantial agreement between μQFR and pressure-derived iwFR as a reference gold standard method. As an angiography-derived index not depending on pressure wire or adenosine, μQFR can be a promising option to overcome some of the mentioned limitations. The use of AI in μQFR analysis has facilitated automatic lumen contouring and frame counting which significantly reduced the analysis time (average 67 ± 22 s), with an anticipated rapid learning and training curve with high reproducibility [18].

In concordance with our results, Wu et al. have reported good correlation (*r* = 0.89) and agreement (mean difference, − 0.008; 95% limits of agreement, − 0.080 to 0.063) between μQFR and FFR in a large retrospective study on 289 vessels [22]. In assessing the diagnostic accuracy of μQFR in the present study, the AUC was 0.84, 95% CI 0.717 to 0.962. In addition, the sensitivity and specificity for detecting functionally significant coronary stenosis were 89% and 74%, respectively, while the PPV and NPV were 70% and 91%, respectively. Wu et al. demonstrated relatively higher AUC (0.95 (95% CI, 0.92–0.98)), similar sensitivity 90%, and NPV 96%, but with higher specificity 97% and PPV 92% [22]. The lower correlation, specificity and PPV demonstrated in our study could be explained by the smaller number of examined vessels in a different patient population.

Ding et al. [23] demonstrated good diagnostic accuracy for μQFR, comparable to 3D-μQFR in identifying functionally significant coronary lesions. Furthermore, μQFR was shown not to be significantly affected by the presence of abnormal cardiac structure, valvular regurgitation, or LV diastolic dysfunction [24]. Interestingly, side branch μQFR < 0.8 was strongly associated with future clinical events in patients with coronary bifurcation lesions treated with provisional approach [25].

In contrast to 3-D QFR assessment which requires 2 angiographic projections with angles of ≥ 25 apart, μQFR analysis needs only single angiographic projection. In some cases, it might be difficult to obtain a second optimal view for accurate three-dimensional reconstruction and QFR calculation, especially in vessels with complex anatomy. Nevertheless, μQFR measurement itself was not feasible in four patients of our study cohort due to vessel overlap, foreshortening, or tortuosity which hindered obtaining an optimal angiographic view needed for accurate μQFR measurement. This represents the most important challenge for computational analysis of μQFR and could explain the expected variability of the results, and the relatively wide confidence interval in the current study.

One vital concern in the present study is the discordance between μQFR and iwFR in 9 (20%) patients for predicting functionally significant coronary lesions. Seven patients (15%) with functionally significant lesions—based on iwFR—were found to be not significant by μQFR. This could be related to the difficulty in getting an adequate angiographic projection to be used for μQFR analysis in some cases. Therefore, the results obtained from μQFR should always be interpreted in the context of the patient symptoms and clinical presentation. Further testing using either invasive wire-based FFR assessment or another noninvasive stress testing may be warranted in some cases when the overall clinical judgment doubts the results of μQFR.

The current study has several limitations. It has the inherent constraint of being a retrospective observational study; however, we included all consecutive patients during the study period to avoid selection bias of patient enrollment. Difficulties in selecting an optimal angiographic view for μQFR analysis in our retrospective angiograms could be mitigated by obtaining the best view with no vessel overlap or foreshortening during prospective coronary angiograms. The number of study patients is also relatively small, because the cost of both iwFR and μQFR hindered including more patients. Future prospective studies with larger cohorts and diverse populations are recommended to validate our study findings.

## Conclusions

µQFR has good diagnostic accuracy for predicting functionally significant coronary lesions with moderate correlation and agreement with the gold standard iwFR. Angiography-derived µQFR could be a promising tool for improving the utilization of physiology-guided revascularization. The prognostic value of µQFR-guided revascularization and its cost effectiveness in low- and middle-income countries should be properly evaluated in a larger prospective study.

## Data Availability

The datasets used and/or analyzed during the current study are available from the corresponding author on reasonable request.

## Abbreviations

FFR: Fractional flow reserve
iwFR: Instantaneous wave-free ratio
QCA: Quantitative coronary angiography
QFR: Quantitative flow reserve
3-D GFR: Three-dimensional quantitative flow reserve
µQFR: Murray law-based QFR
DM: Diabetes mellitus
AI: Artificial intelligence
MLD: Minimal lumen diameter
ROC: Receiver operating characteristics
ACS: Acute coronary syndrome
CCS: Chronic coronary syndrome
ICM: Ischemic cardiomyopathy
CKD: Chronic kidney disease
PCI: Percutaneous coronary intervention
CABG: Coronary artery bypass graft
LAD: Left anterior descending artery
LCX: Left circumflex artery
RCA: Right coronary artery
AUC: Area under the curve
PPV: Positive predictive value
NPV: Negative predictive value
CI: Confidence interval
